# Hnrnpa1 is essential for early zebrafish development and lipid metabolism: insights from a novel zebrafish knockout model

**DOI:** 10.3389/fcell.2026.1789605

**Published:** 2026-06-01

**Authors:** Lara U. Jansen, Özge P. Burhan, Alexander Hruscha, Janina Tokarz, Cornelia Prehn, Alexander Cecil, Jerzy Adamski, Christian Haass, Ting Sun, Stefan Bonn, Bettina Schmid

**Affiliations:** 1 German Center for Neurodegenerative Diseases (DZNE), Munich, Germany; 2 Helmholtz Zentrum München, Neuherberg, Germany; 3 German Center for Diabetes Research, Neuherberg, Germany; 4 Lehrstuhl für Experimentelle Genetik, Technische Universität München, Freising-Weihenstephan, Germany; 5 Department of Biochemistry, Yong Loo Lin School of Medicine, National University of Singapore, Singapore, Singapore; 6 Metabolic Biochemistry, Biomedical Center (BMC), Faculty of Medicine, Ludwig-Maximilians-Universität München, Munich, Germany; 7 Munich Cluster for Systems Neurology (SyNergy), Munich, Germany; 8 Center for Molecular Neurobiology Hamburg (ZMNH), Hamburg, Germany

**Keywords:** HNRNPA1, metabolismn, neurodegeneration, RNA binding protein, zebrafish

## Abstract

RNA binding proteins have multiple diverse cellular functions and are often mis-regulated in disease. Despite their many cellular functions and implications in disease, very little is known about their physiological functions. Here we describe a novel zebrafish knockout model of the RNA binding proteins Hnrnpa1 and Hnrnpa3. Loss of Hnrnpa3 in zebrafish has no obvious morphological phenotype. Similarly, single mutants of the duplicated zebrafish *hnrnpa1* genes, *hnrnpa1a* and *hnrnpa1b*, have no discernible phenotype, whereas the *hnrnpa1a*; *hnrnpa1b* double mutants are embryonic lethal. They display muscle, vascular and developmental defects with a reduced volume of the yolk extension. Metabolic profiling revealed severe changes in lipid metabolism in the *hnrnpa1a; hnrnpa1b* double mutants. Our analysis identified the involvement of Hnrnpa1 in many cellular pathways including the regulation of lipid metabolism and opens the door for future therapeutic studies in HNRNPA-associated diseases.

## Introduction

The heterologous nuclear ribonucleoproteins (HNRNP) proteins are a diverse group of proteins characterized by their ability to bind nucleic acids through RNA recognition motifs (RRM). HNRNPs shuttle between the nucleus and the cytoplasm and form multimeric protein complexes through association with other HNRNP proteins. They fulfill several important functions in nucleic acid metabolism including splicing, translational regulation and RNA transport and stability (Geuens et al., 2016; Krecic and Swanson, 1999). Members of the HNRNP family in humans have been named alphabetically from HNRNPA through HNRNPU when they were first characterized by their ability to bind RNA and to form ribonucleoprotein (RNP) complexes (Dreyfuss et al., 1988; Pinol-Roma et al., 1988).

Mutations in some of the HNRNP family members have been associated with neurodegenerative diseases. The HNRNP family member Tar-DNA binding protein of 43 kDa (TARDBP, TDP-43) for example, is the pathological entity in 97% of amyotrophic lateral sclerosis (ALS) and 45% of frontotemporal dementia (FTD) cases (Taylor et al., 2016; Ling et al., 2013). Mutations in the glycine-rich domain of TDP-43 are associated with familial ALS further supporting its active role in disease (Pesiridis et al., 2009). Furthermore, HNRNPA3 has been identified to bind to the GGCCCC repeats associated with C9orf72 in familial ALS and FTD (Mori et al., 2013) and to be cleared from the nucleus in ALS brains (Neumann et al., 2006). Pathogenic mutations in the glycine-rich domain of the closely related proteins HNRNPA/B and HNRNPA1 are linked to Multisystem Proteinopathy (MSP), a disease characterized by the degeneration of muscle, brain, motor neurons and bone, and to few cases of familial ALS (Kim et al., 2013). TDP-43, HNRNPA1 and HNRNPA3 share the same overall protein domain composition with two RRM domains and a glycine-rich domain through which they physically interact with each other and other RNA binding proteins (Buratti et al., 2005; D'Ambrogio et al., 2009). They have been shown to undergo phase separation (Molliex et al., 2015) to form liquid-liquid droplets to form membrane less cellular compartments. These features suggest that disturbances in the function of TDP-43 and HNRNPA’s, due to mis-localization, aberrant phase separation and aggregation contribute to disease progression in ALS, FTD and MSP (Alberti and Dormann, 2019).

Despite their clear association with disease, the physiological functions of the HNRNP family members are still poorly defined. Our aim is to elucidate the physiological function of HNRNP family members associated with ALS to identify mis-regulated downstream targets that might contribute to disease. We previously generated TDP-43 knockout (KO) zebrafish and describe here the generation and analysis of Hnrpna KO zebrafish. Homozygous *hnrnpa1a*, *hnrnpa1b* and *hnrnpa3* single KO are viable and fertile. However, *hnrnpa1a−/−;*/*hnrnpa1b−/−* double KO are embryonic lethal and display multiple early phenotypes. One of the earliest and most prominent phenotypes is a drastically reduced amount of yolk in the yolk extension of larvae. We hypothesize that this is due to disturbed lipid metabolism, which could potentially lead to neurodegeneration in HNRNPA1 mutation carriers.

## Results

### The HNRNPA family in zebrafish

**FIGURE 1:**
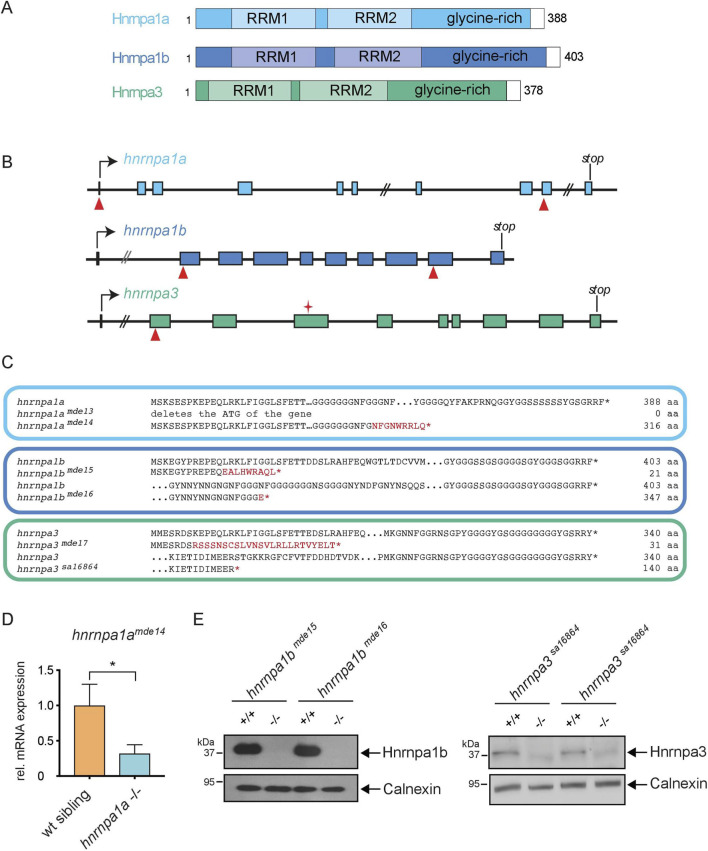
Generation of Hnrnpa1a, Hnrnpa1b and Hnrnpa3 loss of function alleles. **(A)** Schematic representation of zebrafish Hnrnpa1a (turquoise), Hnrnpa1b (blue), and Hnrnpa3 (green) with domain structure. RRM = RNA recognition domain; glycine-rich = glycine-rich domain. White boxes indicate the nuclear localization signal. Numbers indicate the first and last amino acid of the respective protein. **(B)** Scheme of the genomic organization of the *hnrnpa1a*, *hnrnpa1b* and *hnrnpa3* locus. Red arrow heads indicate binding sites for the gRNAs. Symbol in exon3 of *hnrnpa3* indicates location of the *hnrnpa* ^*sa16864*^ mutation. **(C)** Overview of the alleles generated with allele designation. Reference wildtype protein sequence on top. New amino acids generated after the frameshift mutations are indicated in red. Asterisks indicate stop codon. Number at the end of the sequence indicates the predicted length of the respective protein. **(D)** *hnrnpa1a* mRNA levels were measured in *hnrnpa1a* ^*mde14−/−*^ mutants as no Hnrnpa1a specific antibody is available. *hnrnpa1a* mRNA levels are significantly reduced in the brains of *hnrnpa1a* ^*mde14−/−*^ mutants. (*p < 0.02) n = 4. Error bar indicates S.E.M. Normalized to *rflp13a* and *elf1a2*. Student’s t-test. **(E)** Western blot analysis with Hnrnpa1b and Hnrnpa3 specific antibodies. The Hnrnpa1b specific band is absent in brain samples derived from *hnrnpa1b* ^*mde13*−/−^ and *hnrnpa1b* ^*mde14*−/−^ mutants, whereas an Hnrnpa1b specific signal was detected in brain samples of the respective *hnrnpa1b* ^+/+^ siblings. The Hnrnpa3 specific band was absent in all brain samples derived from *hnrnpa3* ^*sa16864*−/−^ mutants, whereas an Hnrnpa3 specific signal was observed in brain samples of *hnrnpa3* ^+/+^ siblings (data shown is from two biological replicates). Calnexin serves as a loading control in both blots.

In humans, the HNRNPA subfamily consists of HNRNPA0, HNRNPA1, HNRNPA1L2, HNRNPA2B2 and HNRNPA3 (Supplementary Figure 1A). HNRNPA0 has 3 orthologues in zebrafish, termed Hnrnpa0a, Hnrnpa0b and Hnrnpa0l. In zebrafish, the *hnrnpa1a* and *hnrnpa1b* gene shows synteny to the human *Hnrnpa1* locus (Supplementary Figures 1A, B) (Postlethwait et al., 1998). In zebrafish, there is no orthologue for the human *HNRNPAB1* gene and one single HNRNPA3 orthologue termed Hnrnpa3 (Supplementary Figure 1B). Overall, the HNRNPA subfamily is well conserved in zebrafish (Figure 1A; Supplementary Figure 1A).

### Generation of *hnrnpa1a, hnrnpa1b* and *hnrnpa3* loss of function mutants

In order to generate loss-of-function (lof) alleles for *hnrnpa1a*, *hnrnpa1b* and *hnrnpa3* in zebrafish we designed gRNAs targeting one of the first exons around the start codon and a second gRNA targeting a downstream exon (Figure 1B). Restriction sites in close proximity to the gRNA target sites were chosen for identification of induced mutations (Supplementary Figures 1C–E). Loss of the restriction site in an PCR amplicon around the gRNA target site was indicative of a positive genome-editing event. At least 2 alleles per gene with a predicted reading frame shift and an early stop codon were selected for further analysis (Figure 1C). For the *hnrnpa3* locus, we additionally analyzed the *hnrnpa3* ^*sa16864*^ allele previously generated by the Wellcome Trust Sanger Institute zebrafish mutagenesis project (Kettleborough et al., 2013). Since there is no specific antibody available against Hnrnpa1a, we performed qRT-PCR analysis and noted severely reduced amounts of *hnrnpa1a* mRNA in the *hnrnpa1a* ^*mde14*^ mutant indicative of a lof allele (Figure 1D). We further confirmed by Western blot analysis that Hnrnpa1b and Hnrnpa3 proteins are undetectable in the respective mutants and that *hnrnpa1b* ^*mde15*^, *hnrnpa1b* ^*mde16*^ *,* and *hnrnpa3* ^*sa16864*^ are lof alleles (Figure 1E).

### Single mutants have no obvious phenotype

**FIGURE 2:**
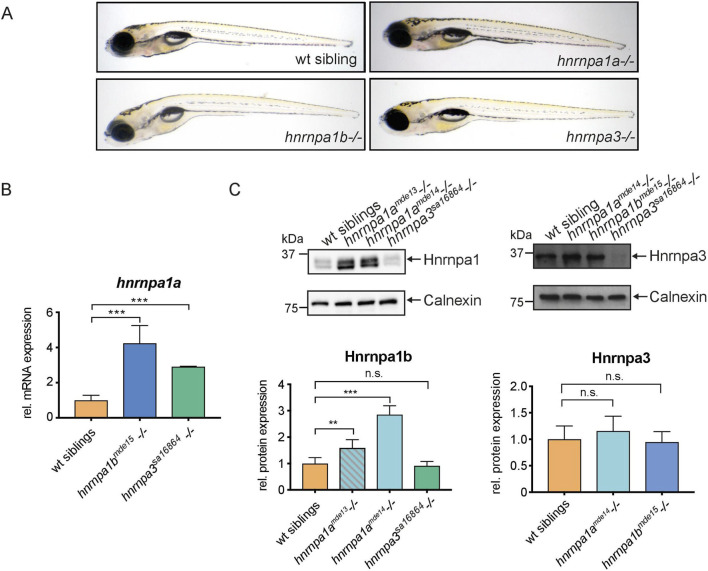
Hnrnpa1a and Hnrnpa1b single KO fish do not show a phenotype due to functional compensation. **(A)** *hnrnpa* ^−/−^ single mutants show no obvious morphological phenotype. Images of 5 dpf old wildtype, *hnrnpa1a* ^*−/−*^, *hnrnpa1b* ^*−/−*^ and *hnrnpa3* ^*−/−*^ mutants. Lateral view. Anterior to the left. Images were taken with Axio Scope A1. **(B)** Hnrnpa1a and Hnrnpa1b but not Hnrnpa3 compensate for each other’s loss. mRNA levels of *hnrnpa1a* were significantly upregulated in brains of *hnrnpa1b* ^*−/−*^ (***p < 0.0001; n = 4) and *hnrnpa3* ^*−/−*^ (***p < 0.0001; n = 4) mutants. Normalized to *rflp13a* and *elf1a2*. Error bar indicates SEM. Unpaired t-test. Results by qRT-PCR were reproduced twice using the same cDNA. **(C)** Hnrnpa1b levels were significantly upregulated in brains of *hnrnpa1a* ^*−/−*^ mutants (*hnrnpa1a* ^*mde13*^: **p < 0.001, n = 8; *hnrnpa1a* ^*mde14*^: ***p < 0.0001, n = 8) and not changed in brains of *hnrnpa3* ^*−/−*^ mutants (*p* > 0.69, n = 4) compared to their wildtype siblings. Hnrnpa3 levels were not changed in brains of *hnrnpa1a* ^*−/−*^ mutants (*p* > 1.1, n = 8) and *hnrnpa1b* ^*−/−*^ mutants (*p* > 0.64, n = 8) compared to their wildtype siblings. Student’s t-test. Error bars indicate S.E.M. Calnexin served as a loading control. Statistical significance was defined as p < 0.05. n.s., not significant.

Upon breeding to homozygosity, we did not observe any obvious morphological larval phenotypes (Figure 2A). All homozygous fish were adult viable and fertile (n > 10). We next investigated muscle and motor neurons, which have been previously described to be affected in Tardbp; Tardbpl dKO mutants (Schmid et al., 2013).

Homozygous *hnrnpa1a*, *hnrnpa1b*, and *hnrnpa3* mutant larvae had wildtype muscle morphology as seen by immunohistochemical staining with muscle specific antibodies (Supplementary Figure S2A). Outgrowing axons of the caudal primary (CaP) motor neuron did not show a reduced length nor any branching defects at 30 h post fertilization (hpf) (Supplementary Figures S2B, C). We speculated that Hnrnpa1a and Hnrnpa1b could potentially act redundantly or cross-regulate each other in zebrafish and mask a potential lof phenotype as previously described for Tardbp and Tardbpl (Schmid et al., 2013; Hewamadduma et al., 2013). Indeed, we found upregulation of *hnrnpa1b* mRNA in Hnrnpa1a mutants and upregulation of Hnrnpa1b protein in Hnrnpa1a mutants, but not in Hnrnpa3 mutants (Figures 2B,C). These findings indicate, that Hnrnpa1a and Hnrnpa1b are part of a feedback mechanism to maintain homeostatic wildtype levels and become upregulated upon loss of the other paralogue.

### Hnrnpa1a/1b double mutants are embryonic lethal

**FIGURE 3:**
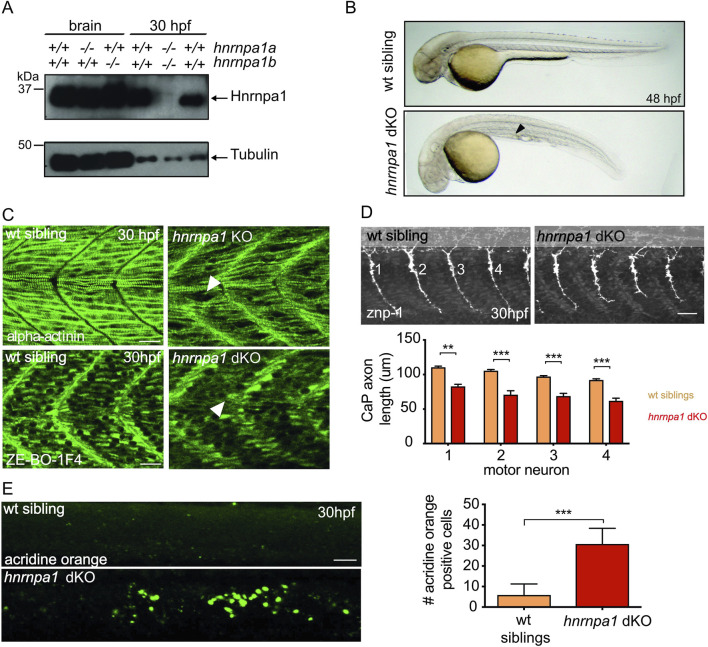
Hnrnpa1 dKO show a lipid, muscle, motor neuron phenotype and increased cell death **(A)** The antibody HA1-CT (VM) AN351 detects Hnrnpa1a and Hnrnpa1b since a band at the expected size of Hnrnpa1a and Hnrnpa1b is detectable in wildtype, *hnrnpa1a* ^*−/−*^ and *hnrnpa1b* ^*−/−*^ zebrafish brains. No band appears at the expected size in *hnrnpa1* dKO embryos 30 hpf further supporting complete loss of Hnrnpa1 protein (overexposed blot to show absence of band in lane 5). Tubulin serves as a loading control. **(B)** After 48 hpf *hnrnpa1* dKO embryos show reduced blood flow and a thinned yolk extension (arrowhead). **(C)** Antibody staining of 30 hpf wildtype and *hnrnpa1* dKO embryos with the α-actinin specific antibody (green). Antibody staining of 30 hpf wildtype and *hnrnpa1* dKO embryos with the myosin specific antibody ZE-BO-1F4 (green). Pictures taken by confocal laser scanning microscopy using the 488 nm laser. Lateral view. Anterior to the left. Scale bar represents 25 µm **(D)** Examples of whole-mount IF stainings of 30 hpf wildtype compared to *hnrnpa1* dKO mutants stained with znp-1 antibody. The four CaP axons anterior to the end of the yolk extension are shown. Images are taken with the spinning disk Cell Observer. Maximum intensity projection. Lateral view. Anterior to the left. Scale bar: 25 µm. A comparison of the CaP axon lengths of wildtype (orange) and *hnrnpa1* KO (red) at 30 hpf revealed reduced CaP axon outgrowth in *hnrnpa1* dKO (**p < 0.001; ***p < 0.0001; n = 16. Error bar indicates S.E.M. Two-way ANOVA. Bonferroni post-test. **(E)** *hnrnpa1* dKO show increased cell death in the spinal cord. Images of wildtype and *hnrnpa1* dKO stained with Acridine Orange (green), which is taken up by apoptotic and necrotic cells (only part of the spinal cord in the trunk is shown). Lateral view. Anterior to the left. Maximum intensity projection of stacks of ten 0.9 µm images. Images were taken with laser scanning confocal microscope using the 488 nm laser. Quantification of Acridine Orange positive cells within the spinal cord of wildtype and *hnrnpa1* dKO. Number of Acridine Orange positive cells is significantly increased in *hnrnpa1* dKO. Error bars indicate S.E.M; n = 9 embryos per experiment; ***p < 0.0001; Student’s t-test. Scalebar represents 40 µm.

**FIGURE 4:**
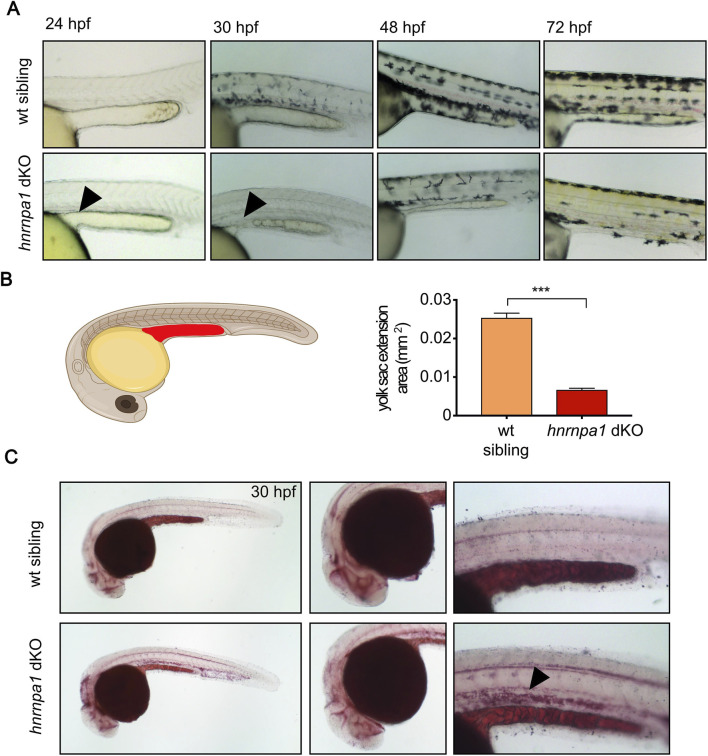
Hnrnpa1 dKOs have lipid metabolism defects. **(A)** *hnrnpa1* dKO embryos have a thinned yolk extension. Images of wildtype (upper panel) and *hnrnpa1* dKO (lower panel) embryos at 24 hpf, 30 hpf, and 48 hpf. At 24 hpf *hnrnpa1* dKO can be hardly distinguished from their wildtype siblings apart from a small bulge in the yolk extension (arrowhead). After 30 hpf the yolk extension of *hnrnpa1* dKO embryos starts to become thin, which becomes more severe over time resulting in a severely reduced yolk extension after 48 hpf up to 72 hpf. Lateral view. Anterior to the left. Images were taken with Axio Scope A1. **(B)** Schematic illustration of the measured area of the yolk extension (indicated in red) at 30 hpf which was used for quantification. Quantification of the two-dimensional yolk extension area at 30 hpf shows significant reduction in yolk extension area in *hnrnpa1* dKO compared to wildtype. Student’s t-test (n = 13; ***p < 0.0001). Error bars indicate S.E.M. **(C)** Oil Red O stained the head structures, the yolk, yolk extension and structures around the spinal cord. Reduced amounts of neutral lipids in the yolk extension are present in *hnrnpa1* dKO embryos at 30 hpf. Additionally, *hnrnpa1* dKO embryos show increased uptake of neutral lipids to the trunk (arrowhead). Lateral view. Anterior to the left. Images were taken with Axio Scope A1 microscope.

To uncover potential Hnrnpa1 phenotypes in zebrafish we analyzed double homozygous *hnrnpa1a* ^*−/−*^ *; hnrnpa1b* ^*−/−*^ embryos (referred from now on as *hnrnpa1* dKO). Western blot analysis with an antibody cross-reacting with Hnrnpa1a and Hnrnpa1b shows a clear signal in *hnrnpa1a* ^*−/−*^ and *hnrnpa1b* ^*−/−*^ adult single mutant brain but is absent in *hnrnpa1* dKO demonstrating that both alleles lack detectable protein levels (Figure 3A). The first visible morphological phenotype is a characteristic progressive thinning of the yolk extension around 24 hpf which becomes more prominent over time (Figures 3B, 4A). Muscle abnormalities are evident around 30 hpf (Figure 3C). The outgrowing axons of the CaP motor neuron are severely reduced in length at 30 hpf indicating either developmental delay or outgrow defects (Figure 3D). Furthermore, there is increased cell death at 30 hpf in the *hnrnpa1* dKOs as indicated by increased acridine orange staining, which labels apoptotic and necrotic cells (Figure 3E). Additionally, we observed impaired blood flow with increased pericardia due to mispatterned intersegmental vessels (ISV) at 30 and 46 hpf (Supplementary Figure 3A). To directly assess if the *hnrnpa1* dKO are developmentally delayed, we measured the head trunk angle at 30 and 48 hpf (Supplementary Figures 3C, D). This angle is getting smaller as the embryo develops and can be used as a direct measure for staging of the early zebrafish embryo. At both timepoints we observed a smaller angle compared to their phenotypically appearing wildtype siblings, indicative of a developmental delay (Supplementary Figures 3C, D). This is supported by a delayed onset of pigmentation in the *hnrnpa1* dKO (Figure 4A). The *hnrnpa1* dKO die during early larval stages and do not reach adulthood.

### Altered lipid distribution in Hnrnpa1 dKO larvae

The thinning of the yolk extension is first observed at 24 hpf by a marked thinning at the border of the yolk and yolk extension (a caudal extension of the yolk) (Figure 4A). The yolk extension is progressively becoming thinner over time and is almost absent at 72 hpf. Quantification of two-dimensional area of the yolk extension (Figure 4B) revealed a significant reduction in size in 30 hpf *hnrnpa1* dKO mutants. The yolk mainly consists of lipids and proteins to provide energy for the developing embryo. In order to assess a potential lipid phenotype, we labeled neutral lipids with Oil Red O (ORO). Staining was detectable in the wildtype embryos in the yolk, the yolk extension, and the head. This lipid staining pattern correlates with previously reported ORO staining (Fraher et al., 2015). In contrast, in *hnrnpa1* dKO strikingly lower levels of lipid were labeled in the yolk extension. In contrast, more lipid staining was observed in the embryo`s body (Figure 4C) suggesting impaired lipid transport and/or metabolism.

### Loss of Hnrnpa1 affects expression of genes associated with lipid metabolism

**FIGURE 5:**
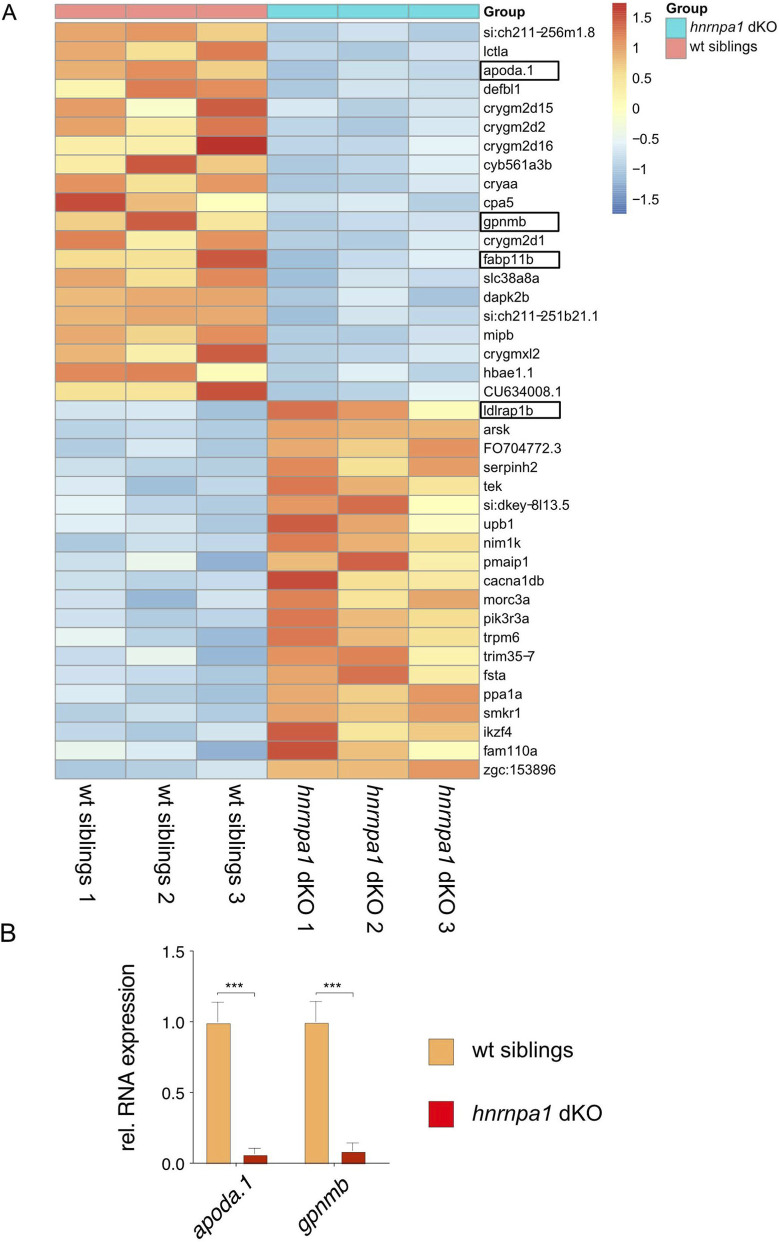
Heatmap of top mis-regulated transcripts in *hnrnpa1* dKO compared to wildtype and qRT analysis. **(A)** Heatmap of the top 20 up and downregulated transcripts in *hnrnpa1* dKO (log2fc <=-1 or ≥ 1 and padj ≤ 0.001). Boxed genes highlight de-regulatd genes in *hnrnpa1* dKO that are associated with lipid metabolism. **(B)** qRT-PCR of *apoda.1* and *gpnmb in* wildtype and *hnrnpa1* dKO. Student’s t-test (n = 13; ***p < 0.0001). Error bars indicate S.E.M.

We next asked what molecular pathways and genes are affected in the *hnrnpa1* dKO and aimed at the identification of the key players leading to the yolk and lipid phenotypes. Bulk RNA sequencing of *hnrnpa1* dKO fish at 30 hpf followed by differential expression analysis (DEG) using DESeq2 (Love et al., 2014) identified 614 differentially regulated genes with more than two-fold change with an adjusted p-value cutoff set to 0.001 (log2fc <=-1 or ≥ 1 and padj ≤ 0.001). 315 of these genes were downregulated and 299 were upregulated (Supplementary Table S1). Of these genes the top 40 hits (log2fc <=-1 or ≥ 1 and padj ≤ 0.001) are shown in a heatmap (Figure 5). Protein-protein interaction and gene set enrichment was done through STRING database with preset default parameters (Szklarczyk et al., 2019), which revealed “cell cycle” as a top cluster, among p53 signaling, foxo signaling, purine metabolism, notch signaling, and others (Supplementary Figure 4; Supplementary Table S2). The findings are consistent with HNRNPA’s role in cancer (Roy et al., 2017) and in line with the delayed development of the dKO zebrafish.

We next validated our sequencing hits by selecting some of the top up and top down hits by qRT-PCR. From the list the downregulated transcripts, the apolipoprotein Da.1 (*apoda.1*) and the transmembrane glycoprotein NMB (*gpnmb*) were also strongly downregulated by qRT-PCR (Figure 5B). From the list of upregulated genes we validated a variety of cell cycle associated hits (*ccne1, cdkn1a, cdkn2a/b, gadd45aa, p53, rbl2*) which were all significantly upregulated, with the exception of *ccne1* (Supplementary Figure 4). Importantly, we also observed a 4.3-fold downregulation of the *hnrnpa1a* transcript and a 2.2-fold downregulation of the *hnrnpa1b* transcript confirming our previous RT-PCR results that the mRNA of the mutant alleles is significantly reduced. The mutant transcripts are not fully absent since mutant mRNA is still transcribed and most likely only partially degraded by non-sense mediated mRNA decay. These findings further validate our RNA sequencing data set.

Importantly, we identified a significant dysregulation in lipid transport proteins. In addition to *apoda.1*, the low density lipoprotein receptor adapter protein 1b and 4a (*ldlpap1b*; *ldlpap4a*) and fatty acid-binding protein 4 and 11b (*fabp7a; fabp11b)* were significantly dysregulated (*ldlpap1b* 2 fold up; *ldlpap4a* 2.2 fold down; *fabp7a* 5.5 fold down*; fabp 11b* 2.6 down). These hits are consistent with a lipid transport phenotype in Hnrnpa1 dKO contributing to the altered lipid distribution and reduced yolk extension phenotypes.

### Metabolic profiling identified prominent lipid changes in *hnrnpa1* dKO

**FIGURE 6:**
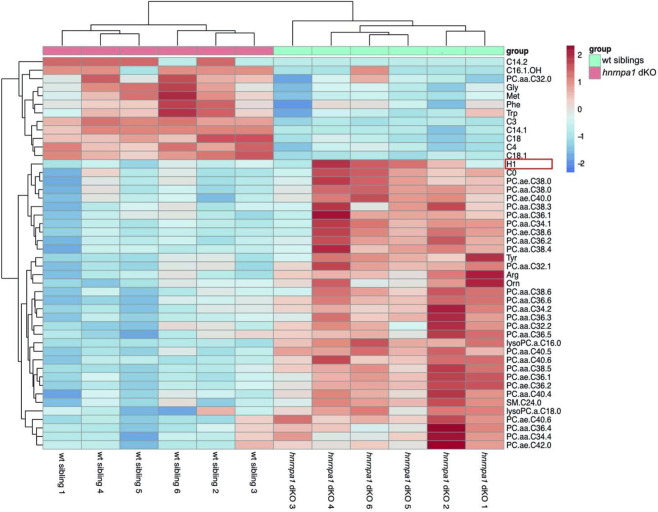
Metabolic analysis of *hnrnpa1* dKO. Heatmap of the top 50 up and downregulated metabolites in *hnrnpa1* dKO. Red box highlights H1 metabolite in *hnrnpa1* dKO.

To determine if *hnrnpa1* dKO suffer from a disturbed metabolism associated with altered lipid distribution, we performed a targeted metabolomics analysis and compared a panel of 180 metabolites in wildtype and *hnrnpa1* dKO (Supplementary Tables S3, 4). The 50 top mis-regulated metabolites are shown in Figure 6A. While there were few amino acids and acylcarnitines (C4, C18, C14:2, C18:1, and others) reduced, we noted a pronounced increase of primarily glycerophospholipids and a few amino acids. Additionally, the sum of hexoses (including glucose) are also increased. Importantly, the ratio of short chain acylcarnitines to free carnitine (C2+3)/C0) and acetylcarnitine to free carnitine C2/C0 were significantly reduced indicative of reduced β-oxidation (Supplementary Figure 5). In summary, loss of Hnrnpa1 leads to reduced β-oxidation consistent with the accumulation and build-up of lipids in the embryo where they cannot be metabolized to ATP.

## Discussion

In humans, the HNRNPA subgroup is divided into HNRNPA1, HNRNPA1/B2, HNRNPA3 and HNRNPA0 (Geuens et al., 2016). This subgroup is only partially conserved in zebrafish. HNRNPA1 is duplicated into Hnrnpa1a and Hnrnpa1b, and no orthologue of HNRNPA1/B2 was identified in zebrafish. We hypothesize that the duplicated Hnrnpa1 genes are either taking over the function of the absent orthologues or that alternatively HNRNPA1/B2 is only required in mammals but not in lower vertebrates such as teleost.

Despite HNRNPA1 being one of the most abundant proteins in a cell (Dreyfuss et al., 1993; Beck et al., 2011), there is still relatively little known about its *in vivo* function. In mice, KO leads to early embryonic lethality due to severe muscle problems (Liu et al., 2017). The same study reports a severe vascular phenotype, heart edema and abnormalities in the dorsal axis and embryonic lethality upon MO knock down of *hnrnpa1b* in zebrafish. In contrast, our analysis of genetic mutants demonstrates that loss of only one of the paralogues *hnrnpa1a* and *hnrnpa1b* does not display any morphological phenotype, due to functional compensation by the other paralogue. Potentially, the MO used in this study knocks down either both zebrafish paralogues or mutant and KO phenotypes are not identical since MO KD fails to be compensated by transcriptional adaptation and elicits a phenotype whereas loss of protein function might be compensated in KO (Rossi et al., 2015; Kontarakis and Stainier, 2020; Sztal and Stainier, 2020). The RNA transcripts of *hnrnpa1a* and *hnrnpa1b* are both reduced in our mutants, consistent with non-sense mediated RNA decay due to the mutation, which is a prerequisite of transcriptional adaptation (Rossi et al., 2015; Kontarakis and Stainier, 2020; Sztal and Stainier, 2020).

Consistent with mammalian HNRNPA1 splicing and RNA regulation, zebrafish Hnrnpa1b has been reported to regulate maternal-to-zygotic transition during early development through regulation of *pri-mir-430* (Despic et al., 2017) and is cooperating with the ribosomal protein Rpl22 in splicing regulation in zebrafish during early development (Zhang et al., 2017). These findings highlight its important and conserved role in splicing regulation across vertebrates (Zhu et al., 2001). We further show that loss of function of Hnrnpa3 does not lead to a morphological phenotype in zebrafish, and no upregulation and thereby potential compensation by *hnrnpa1a* and *hnrnpa1b*. While Hnrnpa1 and Tardbp/Tardbpl (also members of the heterologous nuclear RNA binding protein class) have very dramatic embryonic and larval phenotype and are embryonic lethal in zebrafish, it is surprising to see that Hnrnp3 has no obvious morphological phenotype in zebrafish. In mice, KO of the major isoform HnrnpA3a causes lethality shortly after birth and has been shown to be important for neuronal progenitor cell division (Ou et al., 2020). Future RNA sequencing analysis of the zebrafish *hnrnpa3* mutants will clarify if other RNA binding proteins are able to compensate, the KO phenotypes are subtle or if Hnrnpa3 is non-essential for survival in zebrafish.

RNA sequencing and pathway analysis of Hnrnpa1 dKO zebrafish revealed its prominent role in cell cycle regulation. In humans, HNRNPA1 has been previously shown to be highly upregulated in a large variety of different tumors in humans, including lung cancer, ovarian cancer and colon cancer (Liu et al., 2016; Rodriguez-A et al., 2017; Ushigome et al., 2005; Nishikawa et al., 2019). Regulation of cell cycle therefore is a conserved feature of zebrafish and human HNRNPA1. Mechanisms to convey tumor progression in humans include splicing alterations in key metabolic genes such as pyruvate kinase (Chen et al., 2010), regulation of miRNAs (Rodriguez-A et al., 2017) and modulation of malignant transformation (Roy et al., 2017). Splicing of pyruvate kinase as one of the major factors to promote tumor formation in humans is not altered in zebrafish Hnrnpa1 dKO (data not shown) suggesting different cell cycle regulatory targets (Roy et al., 2017).

More recently mutations in the low complexity domain of HNRNPA1 and HNRNPA2/B1 have been linked to the neurodegenerative diseases multisystem proteinopathy (MSP) and amyotrophic lateral sclerosis (ALS) (Kim et al., 2013; Taylor, 2015). The mutations increase the proteins’ propensity to aggregate in disease and enhance phase separation into membrane-less organelles such as stress granules (Kim et al., 2013; Molliex et al., 2015). These alterations of changed kinetics of membrane-less organelles are thought to impair the cell’s ability to cope with stressors and thereby promote neurodegeneration (Kim et al., 2013; Molliex et al., 2015; Taylor, 2015; Purice and Taylor, 2018). In line with this hypothesis, many other proteins, such as the closely related TDP-43, similarly shows upon disease-associated mutations in its low complexity domain increased propensity to aggregate. Despite their many similarities, loss of TDP-43 and Hnrnpa1 in zebrafish leads to considerable phenotypic differences and distinct RNA seq profiles (data not shown). Loss of Tardbp and Tardbpl in zebrafish does also not lead to a yolk extension and lipid accumulation phenotype as seen in the Hnrnpa1 dKO (Schmid et al., 2013), indicating distinct cellular functions.

Here, we identified the age-associated and neuroprotective factor, ApoD to be downregulated by Hnrnpa1 lof in zebrafish. ApoD belongs to the family of lipocalin proteins and has the ability to bind small hydrophobic molecules and has been shown to play an important function in lipid metabolism, lipid trafficking and confers neuroprotection (Rassart et al., 2020). ApoD is highly upregulated during aging and protects cells from oxidative stress (Dassati et al., 2014; Martinez-Pinilla et al., 2015). ApoD KO mice suffer from neuronal loss in the cortex highlighting its importance in neuronal survival (Dassati et al., 2014). ApoD is upregulated in many neurodegenerative diseases including Alzheimer’s disease, schizophrenia and stroke (Dassati et al., 2014; Bhatia et al., 2019) but downregulated in others such as inclusion body myopathy (Hamann et al., 2017), sporadic cases of ALS (Ranjan et al., 2016). In the Hnrnpa1 dKO we observe increased lipid transport from the yolk to the embryo where it accumulates since it cannot be further processed by ß-oxidation. We hypothesize that downregulated ApoD fails to transport some fatty acids in the embryo to provide energy by β-oxidation since we observe a decrease in some acylcarnitines (C4, C18, C14:2, C18:1, and others), which are required to transport fatty-acids across the mitochondrial membrane for β-oxidation and increased glycerophospholipids.

Additionally, loss of nuclear HNRNPA1 and ApoD downregulation impairs the cellular response to oxidative stress, impairs lipid trafficking and thereby potentially accelerate neuronal cell death in diseases with HNRNPA1 aggregation. Restoration of ApoD levels in ALS and MSP patients might therefore provide a valuable therapeutic approach to increase viability of motor neurons.

In summary, the generation of loss of function models for Hnrnpa1 and Hnrnpa3 in zebrafish with a detailed molecular and metabolomic analysis of the Hnrnpa1 dKO phenotype, uncovered a severely disturbed lipid metabolism and ApoD downregulation with potential implications for neurodegenerative diseases.

## Materials and methods

### Zebrafish

Zebrafish embryos were kept at 28.5 °C and were staged according to Kimmel et al. (1995). The wild-type line AB was used for injections and the wild-type line TFL was used for outcrossing. Adult fish were maintained on a Gemma Micro 300 diet (Skretting). All experiments were performed in accordance with animal protection standards of the German Center of Neurodegenerative Diseases and were approved by the government of Upper Bavaria (ROB-55.2-2532.Vet_02-17-21 Regierung von Oberbayern, Munich, Germany). The following mutant zebrafish lines were generated:

*Hnrnpa1a* ^*mde13*^, *hnrnpa1a* ^*mde14*^, *hnrnpa1b* ^*mde15*^, *hnrnpa1b* ^*mde16*^, *hnrnpa3* ^*mde17*^

The line *hnrnpa3* ^*sa16864*^ has been obtained from the Sanger Center (Kettleborough et al., 2013). Multiple alleles were generated for *hnrnpa1a* and *hnrnpa1b* to exclude possible off-target effects. Initial characterization of all alleles revealed no differences between the two alleles within each genotype. The alleles *hnrnpa1a* ^*mde14*^, *hnrnpa1b* ^*mde16*^ *and hnrnpa3* ^*sa16864*^ were used for all experiments unless otherwise stated. The mutant alleles are deposited at the European Zebrafish Resource Center (https://www.ezrc.kit.edu/).

### gRNAs and identification of induced genomic lesions

gRNAs were designed for the *hnrnpa1a, hnrnpa1b* and *hnrnpa3* locus. gRNA target exon and allele generated are in parenthesis. gRNAs are in capital letters. The PAM motif is highlighted in bold. All sequences are shown in 5′-3′ orientation.

*Hnrnpa1a* exon1 (*hnrnpa1a* ^*mde13*^ *)* ATG​GCG​GGT​GGC​ATT​GCT​GCT​GG

*Hnrnpa1a* exon8 (*hnrnpa1a* ^*mde14*^ *)* GCA​GGA​AAC​TTC​GGA​GGT​GGC​GG

*Hnrnpa1b* exon2 (*hnrnpa1b* ^*mde15*^ *)* CAC​GTG​AGC​CAG​AGC​AGC​TGC​GG

*Hnrnpa1b* exon9 (*hnrnpa1b* ^*mde16*^ *)* GGT​GGT​GGT​GGC​GGC​AAC​AGT​GG

*Hnrnpa3* exon2 (*hnrnpa3* ^*mde17*^ *)* GAG​TCG​CGA​CAG​TAA​GGA​GCC​GG.

Genomic lesions induced by gRNAs were identified by PCR amplification around the gRNA targeted cut site and restriction fragment length polymorphism (RFLP) analysis. PCR amplification of genomic DNA was performed with the following primers (all sequences are shown in 5′-3′ orientation):

*Hnrnpa1a* exon1 forward: CCT​TAT​TTG​GGG​GTA​AAA​ACG​TA

*Hnrnpa1a* exon1 reverse: TAC​CTC​TTT​GGA​CAT​GGC​GG

*Hnrnpa1a* exon8 forward: GGC​GGC​GGC​TAT​GAT​AAC​T

*Hnrnpa1a* exon8 reverse: GCA​TTG​CTC​TGA​ATA​AAC​CAC​TAC​A

*Hnrnpa1b* exon2 forward: CCT​TGG​TTT​GAT​CTC​CGT​TAC​C

*Hnrnpa1b* exon2 reverse: TGT​GTT​TGG​ATC​TTT​CAT​CAC​CT

*Hnrnpa1b* exon9 forward: GGC​AAT​GGA​AAC​TTT​GGA​GGT

*Hnrnpa1b* exon9 reverse: TCA​CGT​CAT​TTA​TGC​CTT​TAG​GA

*Hnrnpa3* exon2 forward: AGC​ATT​ATG​CAA​CAC​ATG​GAG​C

*Hnrnpa3* exon2 reverse: CAC​GCA​GTC​TGT​GAG​TTT​GC.

Amplicon size and wildtype restriction fragment lengths upon digest with respective enzyme (in whole-mount):

*Hnrnpa1a exon2:* amplicon 296 bp (Fnu4HI: 201 bp + 7 bp + 88 bp).

*Hnrnpa1a exon8:* amplicon 396 bp (AciI: 297 bp + 99 bp).

*Hnrnpa1b exon2:* amplicon 346 bp (PvuII: 108 bp + 238 bp).

*Hnrnpa1b exon9:* amplicon 305 bp (Fnu4HI: 126 bp + 179 bp).

*Hnrnpa3 exon2:* amplicon 232 bp (Fnu4HI: 98 bp + 136 bp).

### Immunofluorescence

Embryos were fixed in 4% paraformaldehyde (PFA) overnight at 4 °C. Embryos were washed twice 10 min in PBST (PBS with 0.1% Tween 20) and serially dehydrated 10 min in 25%, 50%, 75%, and 100% (vol/vol) methanol. The embryos were further incubated in 100% methanol overnight at −20 °C. Subsequent rehydration was done for 10 min in 75%, 50%, and 25% methanol, respectively, followed by 3 × 10 min PBST washes. 30 h post fertilization (hpf) old embryos were additionally incubated for 10 min in 1 mg/mL Collagenase. Embryos were then washed 3 × 10 min with PBST and incubated for 1 h in newborn calf serum with 0.1% (vol/vol) Tween 20 (NCST) followed by incubation in the respective primary antibody in NCST at 4 °C. The following day embryos were washed 4 × 30 min with PBST.

### Oil Red O staining

Embryos were treated with 0.03 mg/mL phenylthiourea (PTU) in E3 medium (5 mM NaCl, 0.17 mM KCl, 0.33 mM CaCl2) from 24 h post fertilization at 28,5 °C until fixation. PTU treated embryos were fixed in 4% paraformaldehyde (PFA) overnight at 4 °C. Embryos were washed three times for 10 min in PBST (PBS with 0.1% Tween 20). Oil Red O (ORO) is a fat-soluble lipid dye used to stain lipids. A 5% ORO solution was prepared by dissolving ORO in isopropanol and then diluted to 0.3% ORO in sterile H_2_O and centrifuged for 5 min at 11,000 rpm. 500 μL of the ORO staining solution was added to the embryos and incubated for 1 h at RT on a shaker. The embryos were then washed 3 × 10 min in 1 x PBST and imaged in 1.5% low melting agarose using a Axio Scope A1 microscope.

### Cloning

For all cloning purposes, 2 dpf AB cDNA was used as a template. The following primers were used:

*Hnrnpa1a forward:* CGT​GAC​CGC​CAT​GTC​CAA​AG

*Hnrnpa1a reverse:* ATC​TAA​AAC​CTC​CGT​CCG​CC

*Hnrnpa1b forward:* GTC​GGT​AGG​ATG​TCC​AAA​GAG

*Hnrnpa1b reverse:* TTA​AAA​CCG​TCT​ACC​GCC​AGA​G

*Hnrnpa3 forward:* GCG​CAA​AAG​CTA​CAG​CAT​GG

*Hnrnpa3 reverse:* ACT​TAC​CAC​TCC​AAT​TAA​TCT​GCT

*Apoda.1 forward:* ATG​AAG​GTG​TTT​CTG​GTC​GTG

*Apoda.1 reverse:* TCA​AAG​TTT​TTG​GTC​GCA​TC.

All sequences are shown in 5′-3′ orientation. The PCR products were cloned into the pCR8/GW/TOPO vector and further recombined with LR Clonase II into pCS2+/GW (pCR8/GW/TOPO/TA cloning kit; Invitrogen).

### RNA sequencing

Strand specific, polyA-enriched RNA sequencing was performed as described earlier (Abdel Malik et al., 2017). Briefly, RNA was isolated from whole-cell lysates using the AllPrep RNA Kit (Qiagen) and RNA integrity number (RIN) was determined with the Agilent 2100 BioAnalyzer (RNA 6000 Nano Kit, Agilent). For library preparation, 1 μg of RNA was poly(A) selected, fragmented, and reverse transcribed with the Elute, Prime, Fragment Mix (Illumina). End repair, A-tailing, adaptor ligation, and library enrichment were performed as described in the Low Throughput protocol of the TruSeq RNA Sample Prep Guide (Illumina). RNA libraries were assessed for quality and quantity with the Agilent 2100 BioAnalyzer and the Quant-iT PicoGreen dsDNA Assay Kit (Life Technologies). RNA libraries were sequenced as 150 bp paired-end runs on an Illumina HiSeq4000 platform. On average, about 10.7 Gb of sequence per sample was generated (quality control information is provided in Supplementary Table S1). The STAR aligner (v 2.4.2a) (Dobin et al., 2013) with default parameter settings (except one: -twopassMode = Basic) was used for split-read alignment against the zebrafish genome assembly GRCz11 (Ensembl release 91) and Ensembl gene annotation (both downloaded from Ensembl). To quantify the number of reads mapping to annotated genes we use HTSeq-count (v0.6.0) with default parameter settings (Anders et al., 2015). FPKM (Fragments Per Kilobase of transcript per Million fragments mapped) values are calculated using custom scripts. The output of HTSeq-count was then used as input for the R Bioconductor package DESeq2 (Love et al., 2014) which was used for differential expression analysis following the standard workflow (https://bioconductor.org/packages/release/bioc/vignettes/DESeq2/inst/doc/DESeq2.html). Multiple testing correction was performed using the Benjamini–Hochberg method. Genes were defined as differentially expressed if they exhibited an adjusted *p*-value (FDR) < 0.001 and an absolute log2 fold change ≥1.

### gRNA injections

gRNAs were synthesized *in vitro* using the MEGAshortscript T7 Transcription Kit (Ambion) as described in (Hruscha and Schmid, 2015). An injection mix of 1.5 µL of one gRNA (1-3 μg/μL) and 1.5 µL Cas9 protein (0.5 μg/μL) was prepared and approximately 2 nL of the injection mix was injected into AB eggs to generate the mutant alleles.

### Quantitative RT-PCR

For RNA extraction, pools of 20 fresh-frozen embryos at 30 h post fertilization (hpf) were used. Total RNA was extracted using the RNeasy Kit (Qiagen) and treated with TURBO DNase (Thermo Fisher Scientific). First-strand cDNA synthesis was performed using M-MLV reverse transcriptase (Invitrogen) and random hexamer primers (Fermentas) according to the manufacturer’s instructions. Quantitative real time PCR was performed using SYBR Green (Invitrogen).

The following primers were used:

*Apoda.1 forward:* AAA​ACA​ATT​GAC​GGG​ACG​GC

*Apoda.1* reverse: GCG​TGT​AGG​GCA​AAA​CAT​AGG

*Ccne forward:* ACT​TGC​AGC​TTC​AGC​ACT​CT

*Ccne reverse:* ACC​ACT​TCA​GCC​CTG​AAA​CTT

*Cdkn1a forward:* TCC​CGA​AAA​CAC​CAG​AAC​GA

*Cdkn1a reverse:* TGG​TAG​AAA​TCT​GTG​ATG​TTG​GTC​T

*Cdkn2a/b forward:* CAG​CAG​CCA​CCG​GAA​ACA​TT

*Cdkn2a/b reverse:* TCA​TCA​CCT​GTA​TAG​GCG​TTC​TTC​T

*Gadd45aa* forward: ACT​CGG​TGA​TTA​AGG​CTC​TGG

*Gadd45aa reverse:* TCA​GGG​TCC​ACA​TTG​AGG​GA

*Gpnmb forward:* ACT​TCA​TTA​CAG​ATA​AGA​TTC​CAC​T

*Gpnmb reverse:* CCC​TCT​GAC​AAA​GAT​GTT​TCT​G

*Hnrnpa1a* forward: AAA​GAG​CAA​CAG​ACC​CCT​CG

*Hnrnpa1a* reverse: TGA​CGA​AGC​CAA​ATC​CCC​TC

*p53 forward:* ACT​CAG​GAA​GGT​CAG​TTG​CTG

*p53 reverse:* TAC​GTT​TGG​TCC​CAG​TGG​TG

*rbl forward:* CCG​CTT​CTA​CAA​CCA​CGT​CT

*rbl reverse:* GGA​GTT​TCA​GCC​TGC​CCA​TT.

All samples were run in triplicates and were normalized to the endogenous housekeeping genes that were amplified with the following primer:

*elf1a forward:* AGC​AGC​AGC​TGA​GGA​GTG​AT

*elf1a reverse:* GTG​GTG​GAC​TTT​CCG​GAG​T

*rpl13a forward:* ATT​GTG​GTG​GTG​AGG​TGT​GA

*rpl13a reverse:* CAT​TCT​CTT​GCG​GAG​GAA​G.

Relative mRNA abundance was calculated using the ΔΔC_t_ method. Data were assumed to follow a normal distribution, and statistical analyses were performed using a two-tailed unpaired Student’s *t*-test in GraphPad Prism 7. Quantitative RT-PCR experiments were performed using at least four independent biological replicates per condition, each derived from independent embryo clutches.

### Antibodies

α-Tubulin (Sigma-Aldrich, T6199), WB 1:10.000

α-Actinin (Sigma-Aldrich, A78), IF 1:100.

Calnexin (Stressgen, SPA-860), WB 1:7.000

Myosin (ZE-BO-1F4), IF 1:1

znp-1 (DSJB), IF 1:100

Anti-rabbit IgG, HRP conj. (Promega, W4011), WB 1:10.000

Anti-mouse IgG, HRP conj. (Promega, W4021), WB 1:10.000.

Alexa fluor antibodies (invitrogen), IF 1:100

The following antibodies were generated by the Institute of Molecular Immunology, Helmholtz Center Munich by standard procedures:

Hnrnpa1b Z1A1-2A7 (Hnrnpa1b epitope: MSKEGQPREPEQLR), WB 1:1, rat IgG2c).

Anti-rat IgG2c, HRP conj., WB 1:10.000.

### Western blotting

Embryos and brains were frozen in liquid nitrogen and lysed in 4 x Laemmli buffer by pestle mixing. Lysates were boiled for 5 min at 95 °C while shaking at 800 rpm. Supernatant was loaded after a 5 min spin at 13.000 rpm at room temperature. The equivalent of 0.5–1.0 embryos and approximately 1/10 of one adult brain was loaded per lane on 10% Tris-glycine gels. After electrophoresis, proteins were transferred to PVDF membranes (Millipore). Membranes were blocked for 1 h in PBST with 0.2% I-Block powder (Thermo Fisher Scientific). The primary antibody was incubated in block solution overnight at 4 °C. After washing 4 × 15 min with PBST, the secondary antibody was incubated for 1 h in block solution. Development of the membrane after 6 × 15 min PBST washes was performed using ECL Plus (Amersham). Calnexin or α-tubulin served as loading controls and for normalization, as specified in the figure legends. All Western blots were performed with at least 3 biological replicates.

### Metabolomic profiling

Twenty embryos per sample were collected into homogenization tubes, residual liquid was removed, and the samples were frozen in liquid nitrogen. 80 mg glass beads (0.5 mm, VK-05, PeqLab) and 400 µL ice-cold extraction solvent, a 85/15 (v/v) ethanol/10 mM phosphate buffer pH 7.5 mixture (20 µL per embryo) were added to each tube. The samples were homogenized using a Precellys24 homogenizer equipped with an integrated cooling unit (PeqLab) at −4 °C for three times over 20 s at 5500 rpm with 30 s pause intervals to ensure constant temperature during homogenization. Subsequently, samples were centrifuged at 4 °C and 2300 × g for 5 min and 20 µL of the supernatant were used for metabolite quantification.

The targeted metabolomics approach was based on flow injection-electrospray ionization-tandem mass spectrometry (FIA-ESI-MS/MS) measurements by Absolute*IDQ*™ p150 Kit (Biocrates Life Sciences AG). The assay allows simultaneous quantification of 163 metabolites out of 10 µL plasma, and includes free carnitine, 40 acylcarnitines (Cx:y), 14 amino acids (13 proteinogenic plus ornithine), hexoses (sum of hexoses–about 90%-95% glucose), 92 glycerophospholipids (15 lysophosphatidylcholines (lysoPC) and 77 phosphatidylcholines (PC)), and 15 sphingolipids (SMx:y). The abbreviations Cx:y are used to describe the total number of carbons and double bonds of all chains, respectively (for more details see (Römisch-Margl et al., 2012)). The method of Absolute*IDQ*™ p150 Kit has been proven to be in conformance with the EMEA-Guideline “Guideline on bioanalytical method validation (21 July 2011)” (CHMP CfMPfHU, 2011), which implies proof of reproducibility within a given error range. Sample preparation and mass spectrometric measurements were performed as described by the manufacturer in manual UM-P150. The LODs were set to three times the values of the zero samples (extraction solvent).

The assay procedures of the Absolute*IDQ*™ p150 Kit as well as the metabolite nomenclature have been described in detail previously (Römisch-Margl et al., 2012; Illig et al., 2010). Sample handling was performed by a Hamilton Microlab STAR™ robot (Hamilton Bonaduz AG, Bonaduz, Switzerland) and a Ultravap nitrogen evaporator (Porvair Sciences, Leatherhead, U.K.), beside standard laboratory equipment. Mass spectrometric analyses were done on an API 4000 triple quadrupole system (Sciex Deutschland GmbH, Darmstadt, Germany) equipped with a 1200 Series HPLC (Agilent Technologies Deutschland GmbH, Böblingen, Germany) and an HTC PAL auto sampler (CTC Analytics, Zwingen, Switzerland) controlled by the software Analyst 1.6.2. Data evaluation for quantification of metabolite concentrations and quality assessment was performed with the Met*IDQ*™ software package, which is an integral part of the Absolute*IDQ*™ Kit. Metabolite concentrations were calculated using internal standards and reported in µM. Data was uploaded to metaboanalyst.ca (Pang et al., 2020) and the heat map and the volcano plot were generated with the preselected settings and the following specifications: statistical analysis, missing data evaluation, normalization by sum and FDR adjustment.

### Image acquisition

Images were taken with an Axio Scope A1 microscope (Zeiss) and a Cell Observer spinning disk microscope (Zeiss). Brightness and contrast were adjusted using Fiji.

## Data Availability

The data discussed in this publication have been deposited in NCBI’s Gene Expression Omnibus (Edgar et al., 2002) and are accessible through GEO Series accession number GSE327507 (https://www.ncbi.nlm.nih.gov/geo/query/acc.cgi?acc=GSE327507).

## Supplementary material

SUPPLEMENTARY FIGURE S1 Evolutionary conservation of the Hnrnpa family and localisation of gRNA target sequences in *hnrnpa1a*, *hnrnpa1b*, and *hnrnpa3*. **(A)** Phylogenetic tree with the human HNRNPA (black) and zebrafish Hnrnpa (red) family members. **(B)** Syntheny between human and zebrafish *HNRNPA1 and HNRNPA3.* Human HNRNPA1 is located on chromosome 12. In zebrafish two *hnrnpa1* genes have evolved by genome duplication: *hnrnpa1a* and *hnrnpa1b* that are located on chromosome 11 and chromosome 23, respectively. The neighbouring genes of human *HNRNPA1* are *SMUG1* and *CBX5* at the 5′ site and *NFE2* and *COPZ1* at the 3′ site. In zebrafish *smug1* is located 5′ of *hnrnpa1a*, *cbx5* is located 5′ of *hnrnpa1b*, and *nfe2* is located 3′ of *hnrnpa1b*. **(C)** Schematic illustration of the *hnrnpa1a* genomic exon/intron structure. gRNA3 targets the first coding exon. Induced mutations are identified with the restriction endonuclease (RE) Fnu4HI. gRNA4 targets the ninth coding exon close to the patient mutation site and induced mutations are identified with the RE MnlI. **(D)** Schematic illustration of the *hnrnpa1b* genomic exon/intron structure. gRNA1 targets the second coding exon and induced mutations are identified with the RE PvuII. gRNA2 targets the ninth coding exon close to the patient mutation site and induced mutations are identified with the RE Fnu4HI. **(E)** Schematic illustration of the *hnrnpa3* genomic exon/intron structure. gRNA5 targets the second coding exon and induced mutations are identified with the RE Fnu4HI. Red lines: binding and cut sites of the RE. Purple bold: gRNA target sequence. Bold: PAM motif. Scale bar: 100 bp. Schematic illustrations were generated with http://wormweb.org/exonintron.

SUPPLEMENTARY FIGURE S2
*hnrnpa* ^−/−^ single mutants exhibit no morphological muscle or CaP motor axon defects. **(A)** Antibody staining of 30 hpf wildtype, *hnrnpa1a* ^*−/−*^, *hnrnpa1b* ^*−/−*^, and *hnrnpa3* ^*−/−*^ mutants with the α-actinin specific antibody (green). Antibody staining of 30 hpf wildtype, *hnrnpa1a* ^*−/−*^, *hnrnpa1b* ^*−/−*^, and *hnrnpa3* ^*−/−*^ embryos with the myosin specific antibody ZE-BO-1F4 (green). Pictures taken by confocal laser scanning microscopy using the 488 nm laser. Lateral view. Anterior to the left. Scale bar represents 25 µm. **(B)** Whole-mount IF stainings of 30 hpf wildtype, *hnrnpa1a* ^*−/−*^, *hnrnpa1b* ^*−/−*^, and *hnrnpa3* ^*−/−*^ mutants stained with znp-1 antibody. The four CaP axons anterior to the end of the yolk extension are shown. Images are taken with the spinning disk Cell Observer. Maximum intensity projection. Lateral view. Anterior to the left. Scale bar: 25 μm **(C)** A comparison of the CaP axon length of wildtype (orange), *hnrnpa1a* ^*−/−*^ (turquoise) (*p*(1-4)>0.34, n = 7), *hnrnpa1b* ^*−/−*^ (blue) (*p*(1-4)>0.23, n = 7), and *hnrnpa3* ^*−/−*^ (green) (*p*(1-4)>0.99, n = 7) embryos at 30 hpf revealed no significant difference in the CaP axon length when compared to wildtype. S.E.M. Two-way ANOVA. Bonferroni post-test.

SUPPLEMENTARY FIGURE S3
*hnrnpa1* dKO causes vascular phenotypes and developmental delay **(A)** At 30 hpf the *hnrnpa1* dKO embryos display reduced length of intersomitic vessel outgrowth (ISV) compared to their wildtype siblings but the correct number of sprouts is formed. Scale bar represents 25 µm. Anterior to the left. Lateral view. Maximum intensity projection. Images were taken with Cell Observer spinning disc microscope. At 46 hpf the *hnrnpa1* dKO embryos display vascular mis-patterning reflected by ISV that are misconnected (white arrow) or ISVs that are still unconnected (black arrow). Scale bar represents 25 µm. Anterior to the left. Lateral view. Maximum intensity projection. Images were taken by confocal laser scanning microscopy. **(B)** At 30 hpf the head angle of wildtype embryos was measured at 86° and for *hnrnpa1* dKO mutants at 81°. The reference angle at 30 hpf is 90°. At 48 hpf the head angle of wildtype embryos was measured at 139° and for *hnrnpa1* dKO mutants at 121°. The reference angle at 48 hpf is 138°. Lateral view. Anterior to the left. Images taken by Axio Scope A1 microscope. **(C)** The head trunk angle is significantly smaller in *hnrnpa1* dKO mutants at 30 hpf (*p* < 0.02; n = 6) and 48 hpf (*p* < 0.02; n = 6) compared to age matched wildtype. Student’s t-test. Error bar indicates S.E.M.

SUPPLEMENTARY FIGURE S4 GO enrichment analysis of DEGs in *hnrnpa1* dKO and qRT-PCR verification of upregulated genes involved in cell cycle regulation. GO enrichment analysis of DEGs (FDR ≤ 0.001), shown separately for biological process **(A)**, molecular function components **(B)** and KEGG pathway analysis **(C)**. Bar length and label indicate the number of genes belonging to respective GO groups. **(A)** GO enrichment analysis of DEGs (FDR ≤ 0.001) biological process (left panel). mRNA expression of the cell-cycle related transcripts *cdkn1a*, *cdkn2a/b*, *gadd45*, *p53*, and *rbl2* are increased in *hnrnpa1* KO mutants. Relative mRNA expression of *cdkn1a* (***p* < 0.004), *cdkn2a/b* (****p* < 0.0004), *gadd45aa* (***p* < 0.005), *p53* (****p* < 0.0003), and rbl2 (****p* < 0.0007) but not *ccne* (*p* > 0.40) is increased in *hnrnpa1* KO (red) compared to their wildtype siblings (orange). n = 4 pools of embryos of independent clutches at 30 hpf. Student’s t-test. Results by qRT-PCR were reproduced twice using the same cDNA. Error bars indicate S.E.M.

SUPPLEMENTARY FIGURE S5 Vulcano plot of the metabolite ratios. Metabolite ratios from wildtype compared to *hnrnpa1* dKOs displayed in a volcano plot (ratios determined by Met*IDQ* ^TN^ RatioExplorer listed in Table 3). Significantly altered ratios are (C2+C3)/C0 ratio of short chain acylcarnitines to free carnitine (p = 0.04) and C2/C0 ratio of acetylcarnitine to free carnitine (p = 0.08).

SUPPLEMENTARY TABLE S4 List of metabolites measured with the Absolute*IDQ*® p150 Kit GAC, Helmholtz Zentrum München.
