# Supplementary figures and images for: Hnrnpa1 is essential for early zebrafish development and lipid metabolism: insights from a novel zebrafish knockout model
Source: Front Cell Dev Biol. 2026 Jun 1;14:1789605. doi: 10.3389/fcell.2026.1789605 (PMC13265542; doi:10.3389/fcell.2026.1789605)

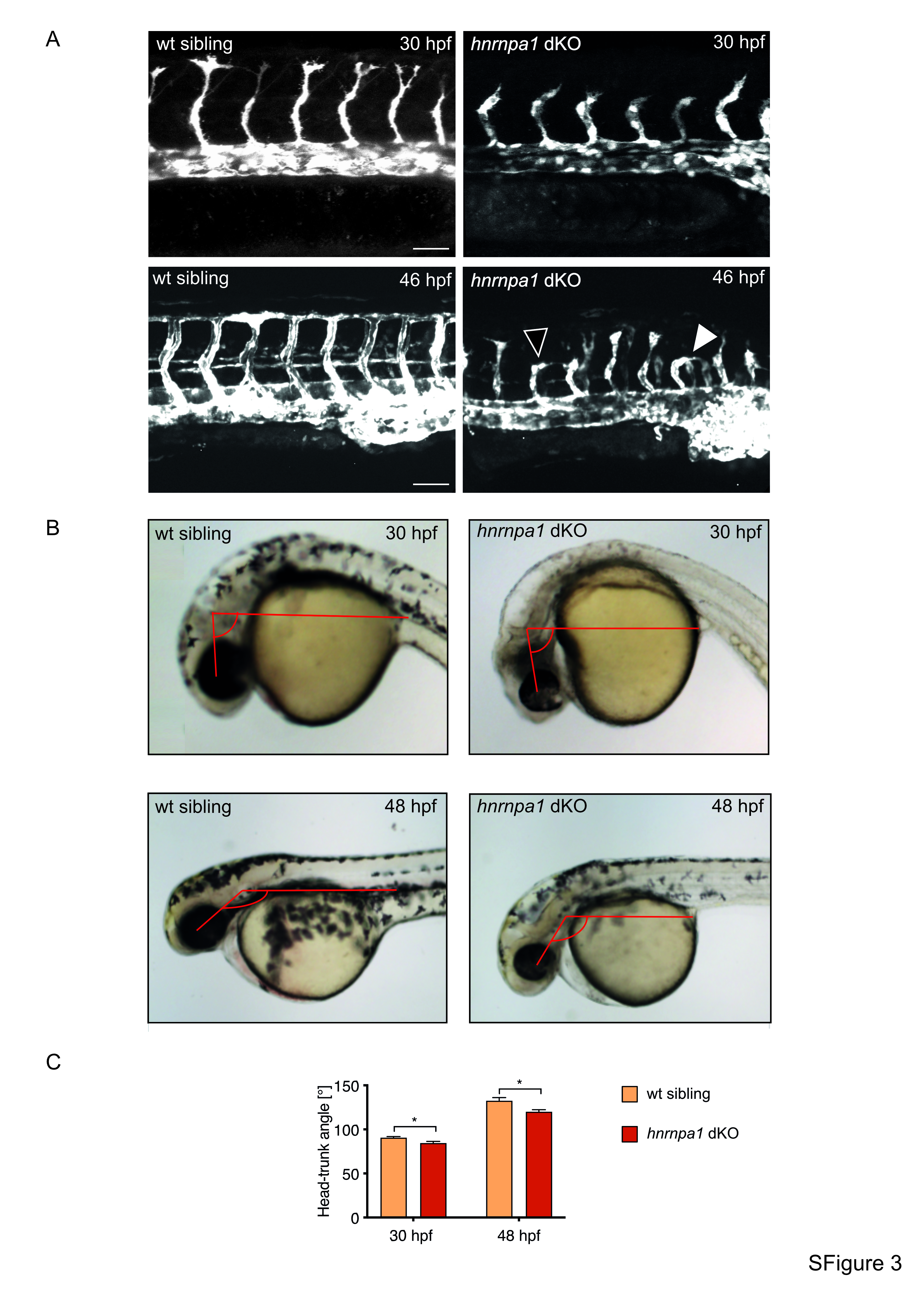

Supplement: Supplementary file 2 [file Image3.jpeg]

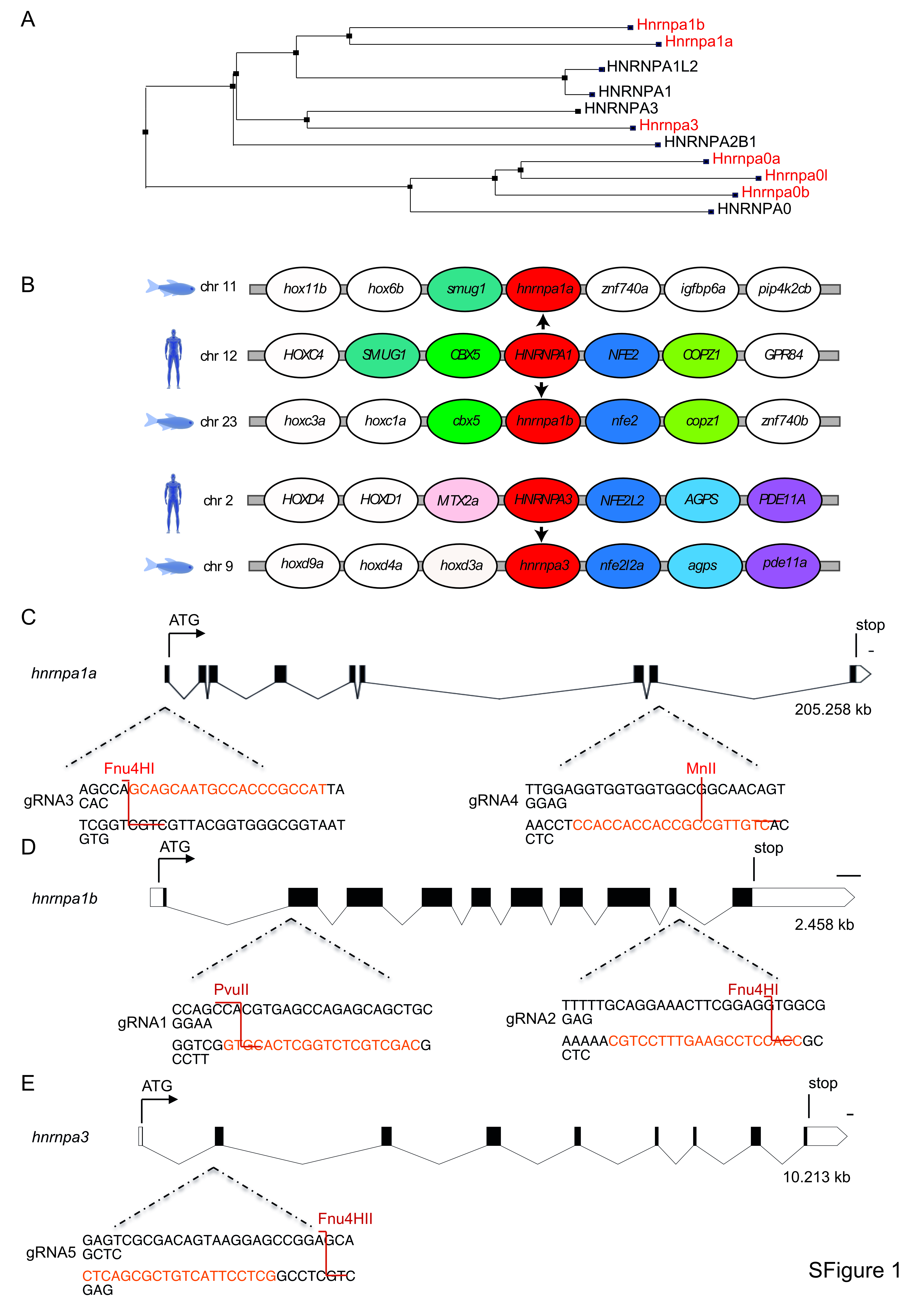

Supplement: Supplementary file 3 [file Image1.jpeg]

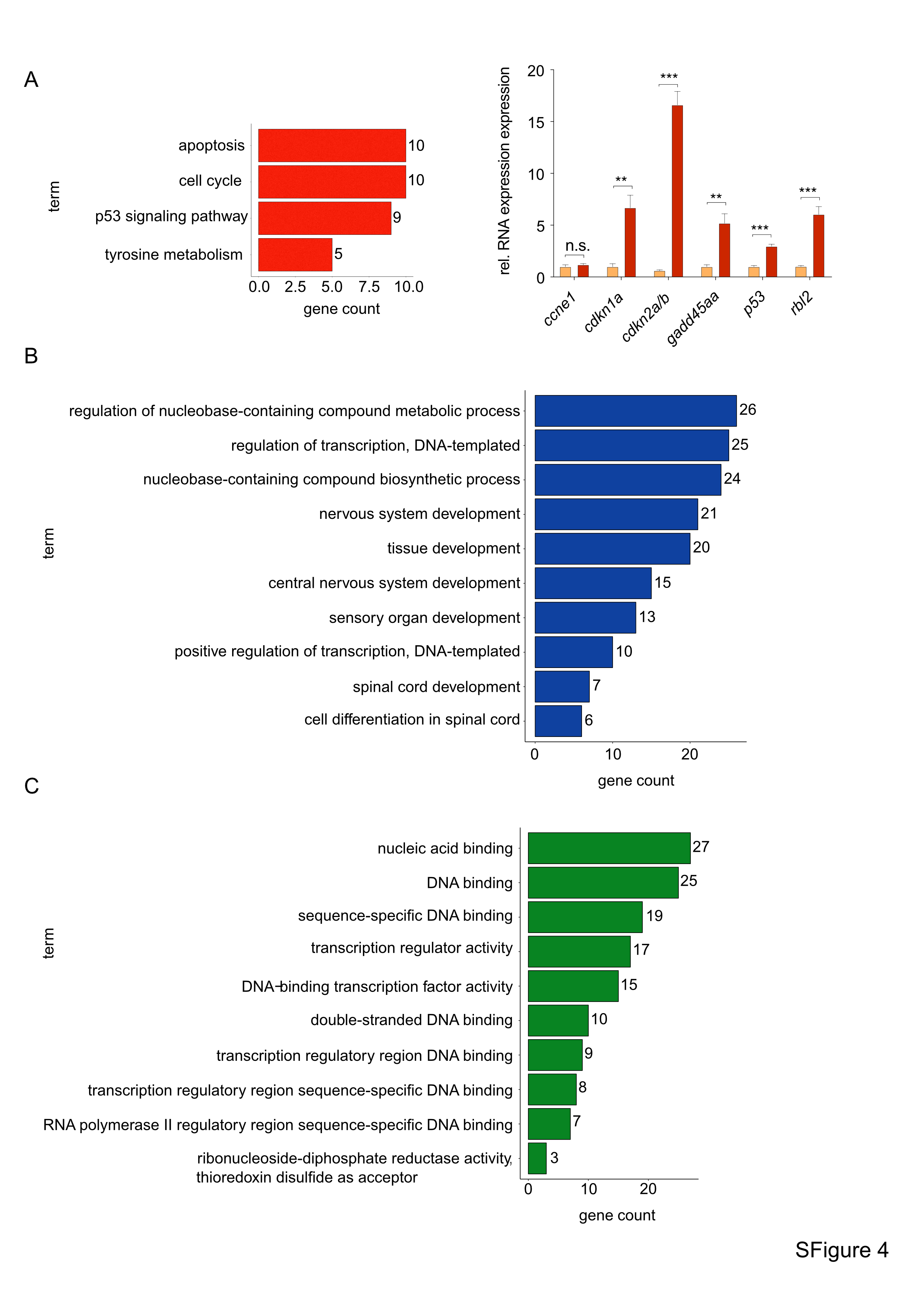

Supplement: Supplementary file 4 [file Image4.jpeg]

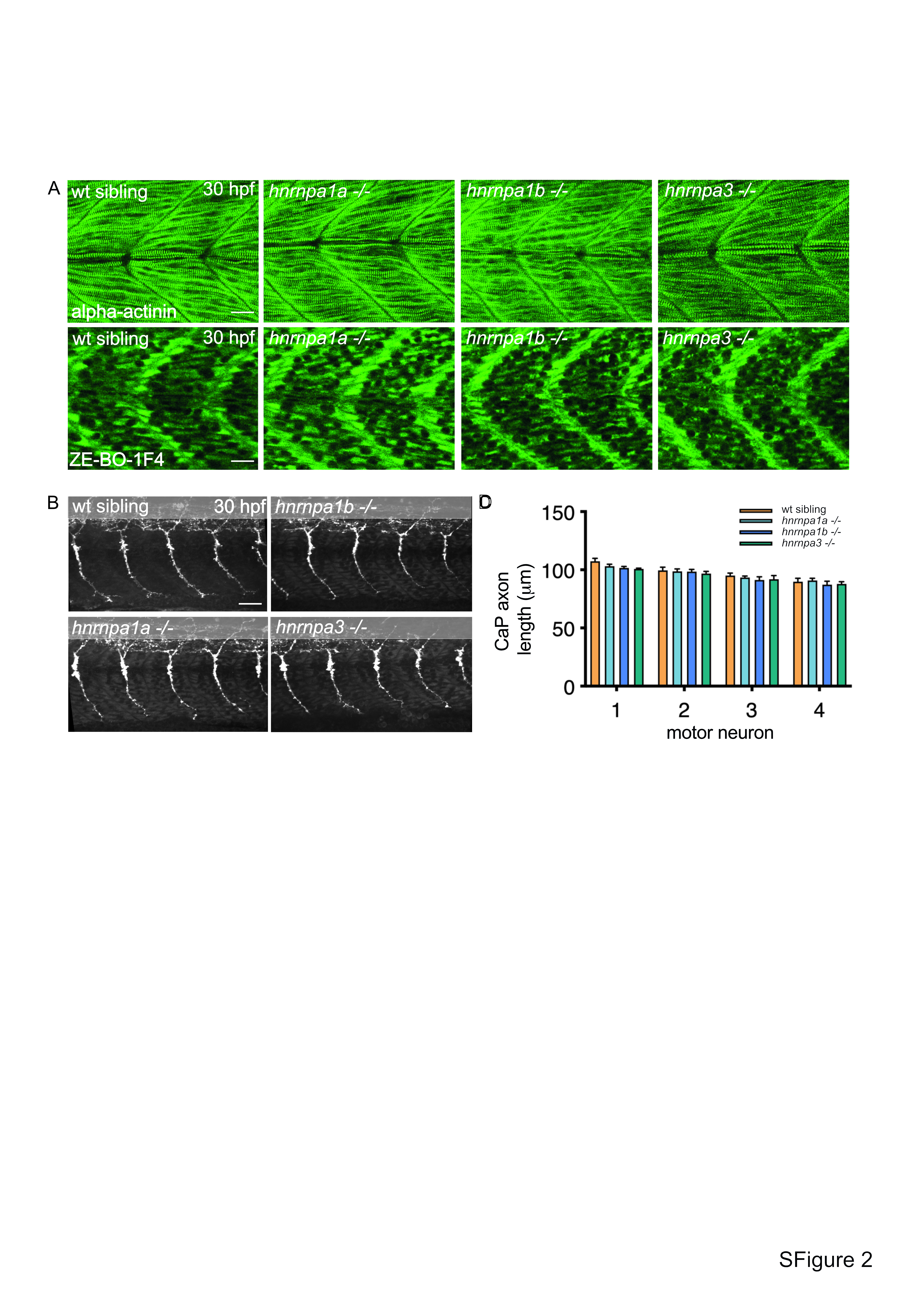

Supplement: Supplementary file 5 [file Image2.jpeg]

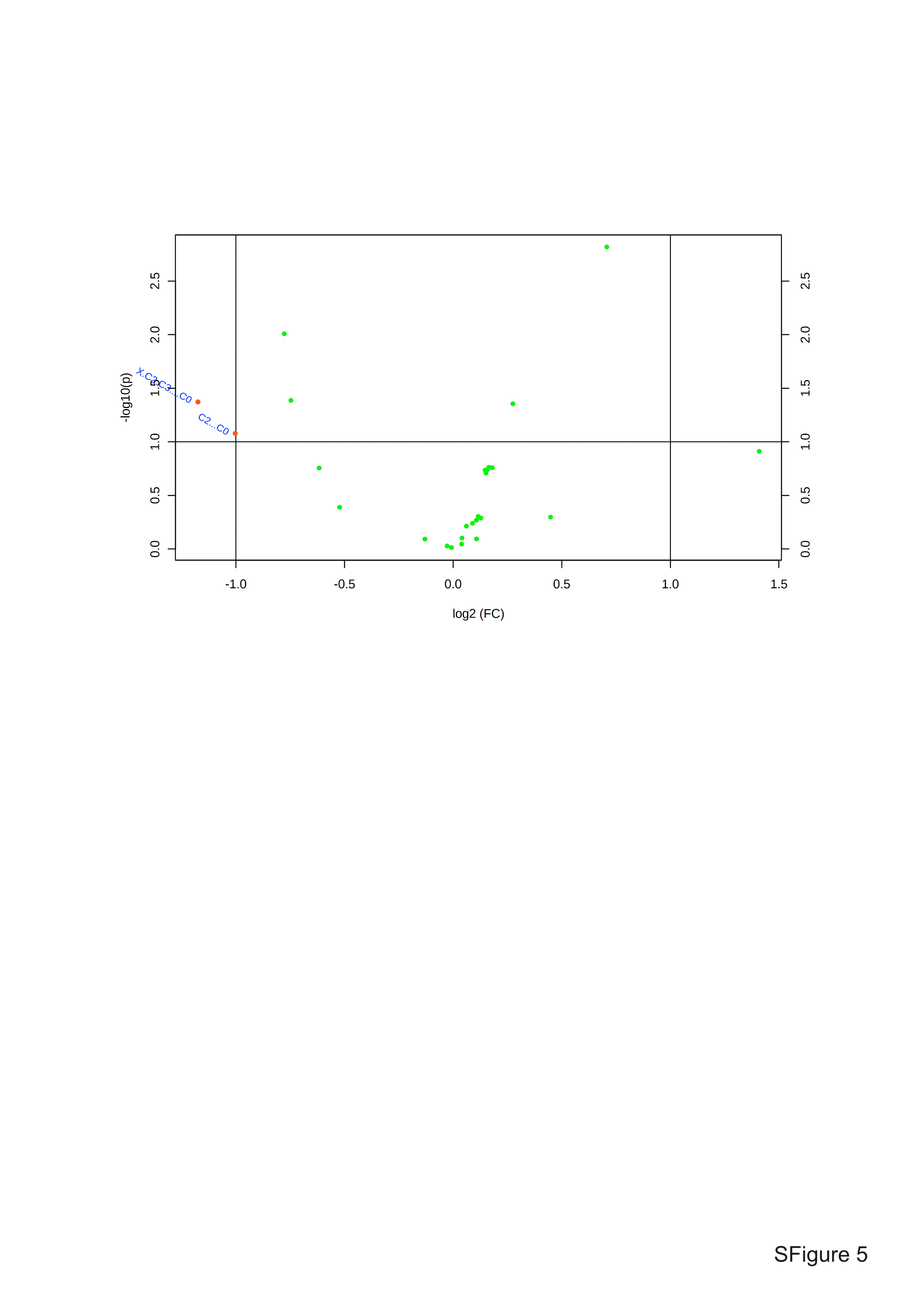

Supplement: Supplementary file 6 [file Image5.jpeg]
